# Spinal intramedullary schwannomas—report of a case and extensive review of the literature

**DOI:** 10.1007/s10143-020-01357-5

**Published:** 2020-09-15

**Authors:** V. M. Swiatek, K.-P. Stein, H. B. Cukaz, A. Rashidi, M. Skalej, C. Mawrin, I. E. Sandalcioglu, B. Neyazi

**Affiliations:** 1 Department of Neurosurgery, Otto-von-Guericke University, Magdeburg, Germany; 2 Department of Neuroradiology, Otto-von-Guericke University, Magdeburg, Germany; 3 Department of Neuropathology, Otto-von-Guericke University, Magdeburg, Germany

**Keywords:** Schwannoma, Spinal tumour, Intramedullary tumour, Review of the literature

## Abstract

Intramedullary schwannomas (IMS) represent exceptional rare pathologies. They commonly present as solitary lesions; only five cases of multiple IMS have been described so far. Here, we report the sixth case of a woman with multiple IMS. Additionally, we performed the first complete systematic review of the literature for all cases reporting IMS. We performed a systematic review of the literature in PubMed, EMBASE and Cochrane Central Register of Controlled (CENTRAL) to retrieve all relevant studies and case reports on IMS. In a second step, we analysed all reported studies with respect to additional cases, which were not identified through the database search. Studies published in other languages than English were included. One hundred nineteen studies including 165 reported cases were included. In only five cases, the patients harboured more than one IMS. Gender ratio showed a ratio of nearly 3:2 (male:female); mean age of disease presentation was 40.2 years; 11 patients suffered from neurofibromatosis (NF) type 1 or 2 (6.6%). IMS are rare. Our first systematic review on this pathology revealed 166 cases, including the here reported case of multiple IMS. Our review offers a basis for further investigation on this disease.

## Introduction

Within the group of central nervous system tumours, spinal tumours represent a minor fraction of 15% of all cases [1]. Spinal schwannomas represent about 10% of all spinal tumours [1]. Schwannomas occur most frequently within the intradural-extramedullary compartment [1]. The intramedullary location of schwannomas is a rare condition (0.3–1.5%) [2–4]. Furthermore, they commonly present as solitary lesions. To date, only five cases of multiple intramedullary schwannomas (IMS) have been described [5–9].

Here, we report a 6th case of a female patient with histologically proven IMS of the cervical spinal cord and an additional small lumbar localized lesion. Additionally, we performed the first complete systematic review of the literature searching PubMed, EMBASE and Cochrane Central Register of Controlled Trials (CENTRAL) for all cases reporting IMS.

### Case report

A 53-year-old woman presented with a 4-month history of progressive sensory deficits of the upper and lower limbs, without any further neurological symptoms. There were no neurofibromatosis (NF) stigmas and no history of genetic disorders or spinal injury.

#### Clinical presentation

Neurological examination revealed hypaesthesia of the first three fingers of the right hand, the right lateral lower leg and the right lateral foot edge. There was no paresis of the upper and lower limbs; the muscular tension was normal. The muscle stretch reflexes were normal and symmetrical. No pyramidal tract signs were present, nor spinal ataxia. The patient was defined as grade I according to the modified McCormick scale [10, 11].

#### Imaging findings and additional diagnostics

**Fig. 1:**
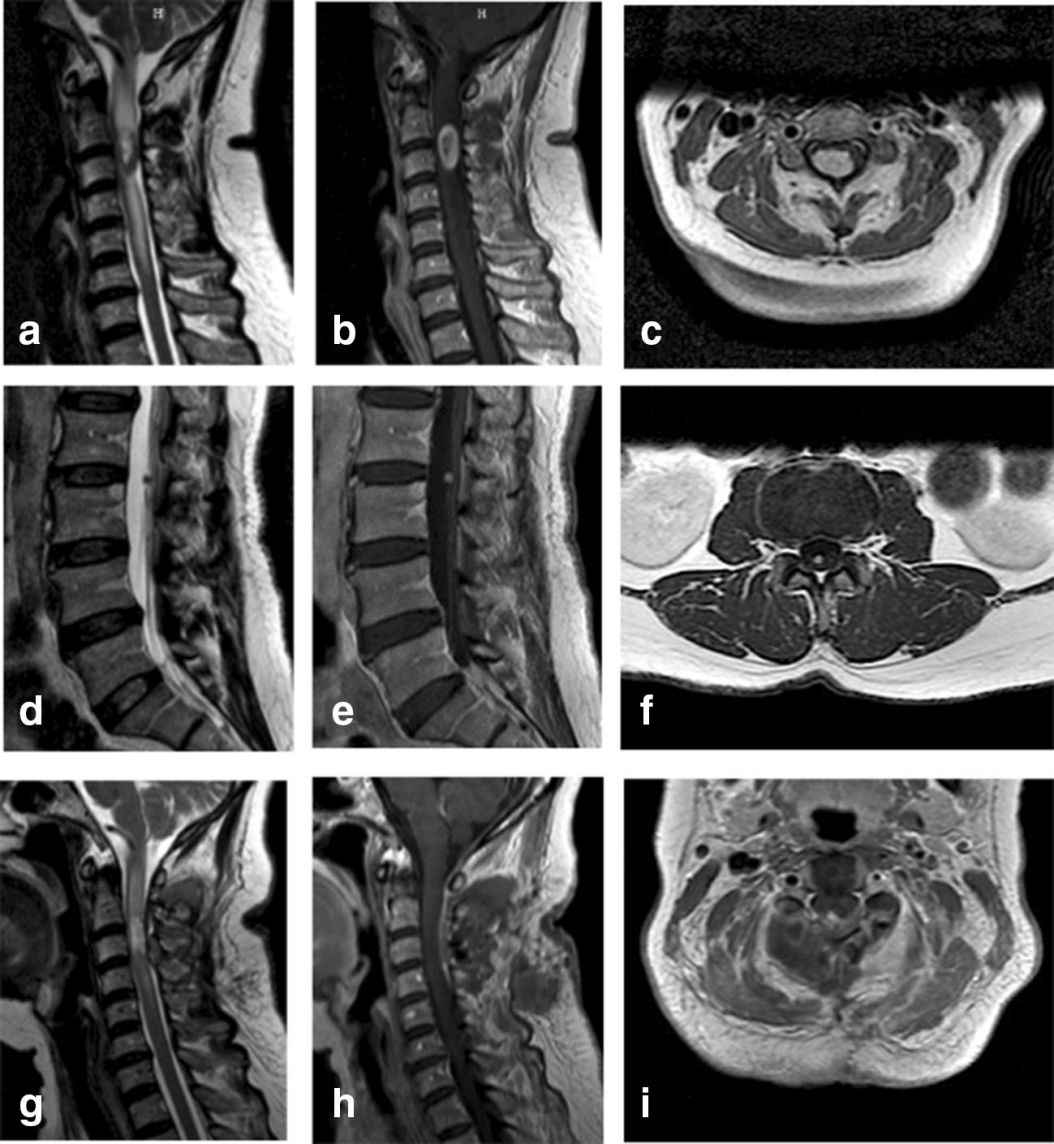
a–c Preoperative MRI of the cervical spine in sagittal (a, b) and transverse (c) slides. T_2_-weighted images show a hypo- and hyperintense intramedullary lesion at the level of C2/3 (a). T_1_-weighted images show a heterogeneous gadolinium-enhanced tumour in the sagittal (b) and transverse (c) slides. d–f Preoperative MRI of the lumbar spine in sagittal (d, e) and transverse (f) slides. T_2_-weighted images show a hypointense lesion at the level of L2/3 (d). T_1_-weighted images show a homogenous gadolinium-enhanced tumour in the sagittal (e) and transverse (f) slides. g–i Postoperative MRI of the cervical spine in sagittal (g, h) and transverse (i) slides confirming the complete tumour resection

Magnetic resonance imaging (MRI) of the neurocranium and the cervical spine revealed a 9.3 × 19 mm intramedullary lesion at the level of C2/3, which was isointense on T_1_-weighted and had both hypo- and hyperintense components on T_2_-weighted images. The lesion showed intense heterogenous contrast enhancement and caused a massive perilesional spinal cord edema extending from the medulla oblongata to the level of C6 (Fig. 1).

Combining the MRI findings and the neurological examination, we considered a preliminary diagnosis of intramedullary ependymoma. As a consequence, further investigations including a holospinal MRI and a lumbar puncture were carried out to examine the possible presence of drop metastasis. The holospinal MRI revealed a second small (3.4 × 4 mm) lesion at the level of L2/3. The lesion was isointense on T_1_-weighted and hypointense in T_2_-weighted images with homogenous contrast enhancement (Fig. 1). Cerebrospinal fluid examination showed no evidence of atypical, potentially malignant cells.

#### Operative findings and histopathology

The patient underwent uneventful microsurgical tumour resection through a posterior cervical approach and midline myelotomy with subsequent C2–C3 laminoplasty. Intraoperatively, the tumour appeared as a solid, yellowish mass comparable with a schwannoma. Complete tumour resection was achieved via meticulous microsurgical technique and ultrasonic aspiration. Intraoperative monitoring (somatosensory-evoked potentials) remained stable during the entire surgical procedure.

**Fig. 2:**
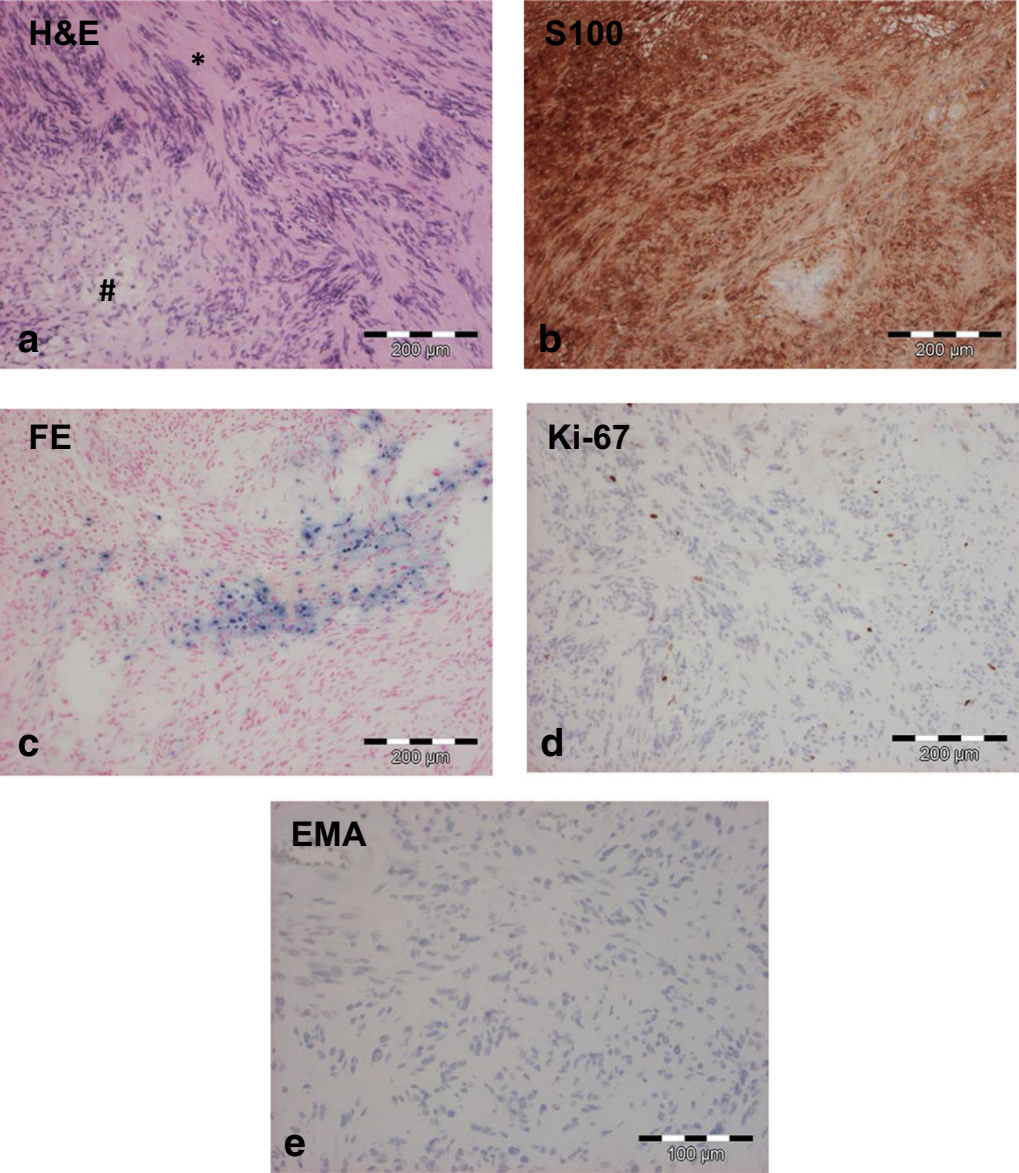
Predominantly spindle cell tumour with fascicular (*) and small myxoid (#) areas (a). Strong immunopositivity for S-100 (b). Old haemorrhages in the tumour (c). Low proliferation rate of the tumour (Ki-67, d). Complete absence of EMA staining in the tumour (e)

Microscopic examination of tissue samples obtained during surgery showed spindle-shaped cells, arranged in a typical fascicular pattern. Small areas consisted of a hypocellular myxoid structure. Old haemorrhages were frequently seen. Immunohistochemistry revealed a strong homogenous reaction for S-100 protein but was negative for epithelial membrane antigen. The proliferation rate (Ki-67 staining) was low (Fig. 2). Altogether, these findings were consistent with a histopathological diagnosis of a schwannoma.

#### Postoperative recovery

Immediately after the surgery, the sensory and motor functions of the patient were intact. During the inpatient stay, the patient had a veritable postoperative course; the sensory impairments remained unchanged. Postoperative MRI of the cervical spine confirmed complete removal of the intramedullary lesion. Interestingly, the massive spinal cord edema decreased almost completely within 10 days after surgery (Fig. 1). The patient was discharged to medical rehabilitation. Follow-up examination 4 months after surgery revealed favourable, unchanged neurological condition (modified McCormick scale: grade I).

## Material and methods

**Fig. 3:**
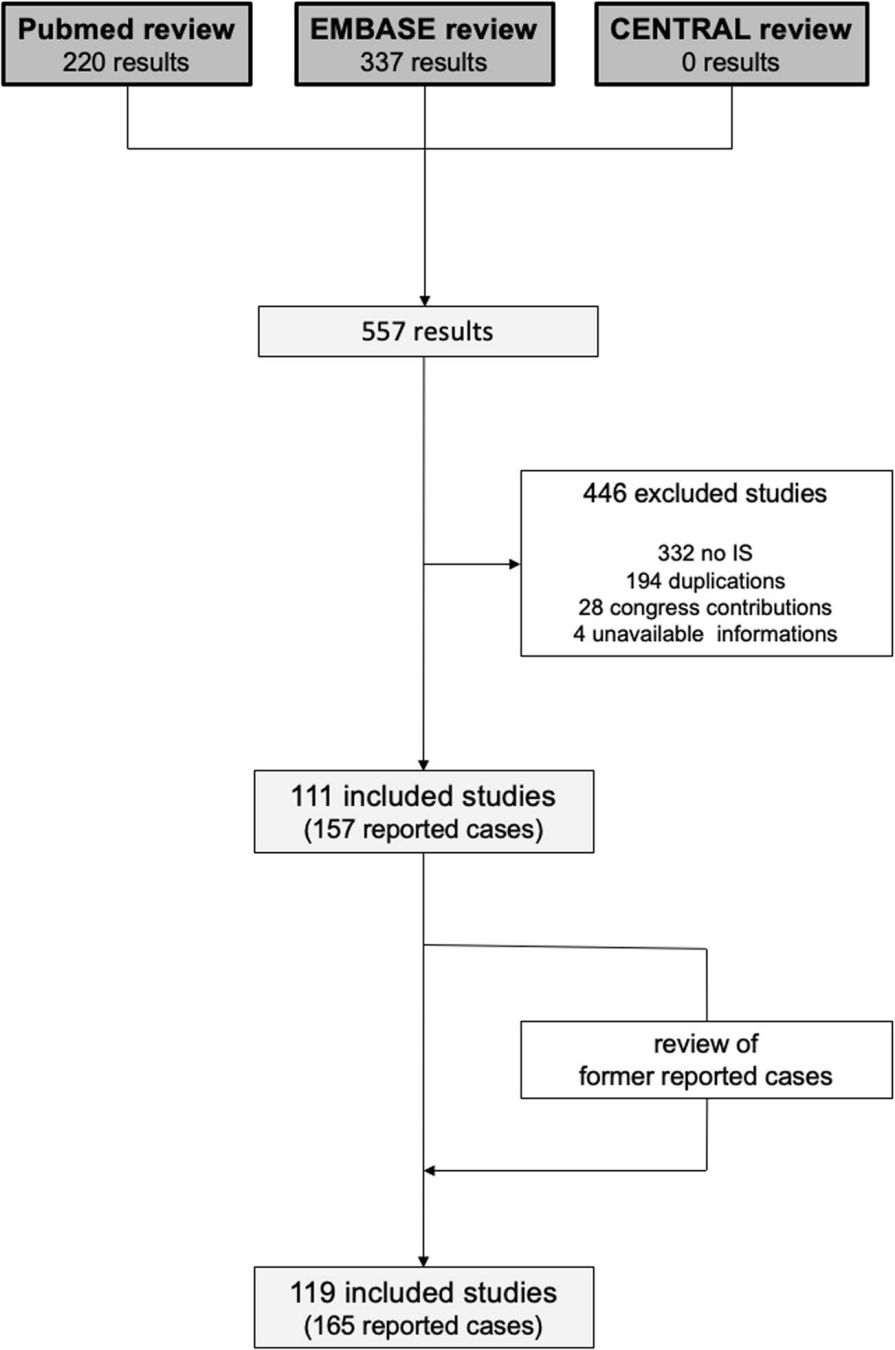
Workflow of the systematic review of the literature

For this study, no experiments on human subjects or animals have been carried out. We performed a systematic review of the literature in PubMed, EMBASE and CENTRAL up to January 1, 2020, to retrieve all relevant studies and case reports on IMS. We used the keywords “intramedullary” simultaneous with “schwannoma OR neurinoma”. Selection criteria were the following: (1) at least one histological proven IMS reported, (2) available clinical information of the patient and (3) peer reviewed publication in a journal or book chapter. Studies published in other languages than English were included in order to receive a complete review of all reported cases. Melanotic IMS were excluded because of their reclassification as a distinct entity in 2016 [12]. In a second step, for complete identification, all reported studies on IMS have been analysed regarding additional cases of IMS. Each case which was mentioned in these articles was analysed with respect to our inclusion criteria. If not already found via keyword search, the case was added to our systematic review (Fig. 3).

## Results

One hundred nineteen studies including 165 reported cases met our inclusion criteria. In only five cases, the patients harboured more than one IMS. Gender ratio was nearly 3:2 (male: female; 55.4% male; 39.2% female); mean age of disease presentation was 40.2 years (range 1 day–78 years); eleven patients suffered from NF (6.6%). A closer analysis of patients suffering from NF revealed that one patient had NF type 1, eight patients had NF type 2 and in two cases no information on the NF type was available. Most IMS were located in the cervical (45.8%) and thoracic (37.3%) spine; a smaller number was located in the cervicothoracic (6.2%), thoracolumbar (5.6%) and lumbar (2.3%) spine (Table 1).

**Table 1:** Patients’ characteristics, preoperative neurological status, postoperative outcome and follow-up

Case No.	Reference	Patient			Localization	Symtpoms				OP	Recovery	McCormick scale*		Follow-up		
		Age	Sex	NF	Vertebra	Sensorysystem	Motorsystem	Autonomicnervous system	Duration(months)			PräOP	PostOP	Months	McCormickscale*	Tumourrecurrence
1	Penfield, 1932 [13]	12	M	No	C5	Yes	Yes	No	96	Yes	n.a.	n.a.	n.a.	n.a.	n.a.	n.a.
2	Rasmussen et al., 1940 [14]	12	M	No	C4–7	n.a.	n.a.	n.a.	48	Yes	–	n.a.	n.a.	n.a.	n.a.	n.a.
3	Roka, 1951 [15]	30	M	No	Cerv.	n.a.	n.a.	n.a.	36	Yes	n.a.	n.a.	n.a.	n.a.	n.a.	n.a.
4	Rose, 1954 [16]	61	M	NF 1	C5	n.a.	n.a.	n.a.	n.a.	Yes	n.a.	n.a.	n.a.	n.a.	n.a.	n.a.
5	Riggs/Clary, 1957 [17]	60	M	No	C4/5	Yes	Yes	Yes	n.a.	Yes	–	IV	IV	24	n.a.	No
6	Ramamurthi et al., 1958 [18]	35	M	No	T2	Yes	Yes	Yes	9	Yes	+	V	III	48	III	Yes
7	Scott/Bentz, 1962 [19]	46	F	No	T3	Yes	Yes	No	144	Yes	o	V	V	n.a.	n.a.	n.a.
8	McCormick et al., 1964 [20]	62	M	No	L2	No	No	No	n.a.	No (Autopsy)	n.a.	n.a.	n.a.	n.a.	n.a.	n.a.
9	Sloof, 1964 [9]	62	F	No	Cerv.	Yes	No	No	n.a.	No (Autopsy)	n.a.	n.a.	n.a.	n.a.	n.a.	n.a.
					Cerv.											
					Cerv.											
10	Mason/Keigher, 1968 [21]	37	M	No	T8–10	Yes	Yes	No	3	Yes	+	III	III	6	II	No
11	Chigasaki/Pennybacker, 1968 [22]	75	F	No	T3	Yes	Yes	No	7	Yes	–	V	V	6	n.a.	n.a.
12	Van Duinen, 1971 [23]	24	F	No	C3	Yes	Yes	Yes	48	Yes	+	III	IV	3	II	No
13	Fabres et al., 1972 [24]	26	M	No	T2/3	Yes	Yes	No	13	Yes	+	IV	IV	n.a.	n.a.	n.a.
14	Cambier et al., 1974 [25]	60	M	No	C2–4	Yes	Yes	No	6	Yes	–	III	IV	17	IV	No
15	Wood et al., 1975 [26]	48	M	No	C3	Yes	Yes	No	3	Yes	–	IV	IV	0	n.a.	n.a.
16	Schmitt, 1975 [27]	68	M	No	L1	Yes	Yes	No	n.a.	No (autopsy)	n.a.	n.a.	n.a.	n.a.	n.a.	n.a.
17	Isu et al., 1976 [28]	30	F	No	C1	Yes	Yes	No	6	Yes	n.a.	III	n.a.	n.a.	n.a.	n.a.
18	Kumar/Gulati, 1977 [29]	24	F	NF	Cerv.	Yes	Yes	No	12	Yes	o	V	V	n.a.	n.a.	n.a.
					T7–9											
19	Vailati et al., 1979 [30]	40	F	No	T8/9	No	Yes	No	12	Yes	+	IV	IV	6	II	No
20	Gegalian, 1979 [31]	37	F	No	T10/11	Yes	Yes	No	n.a.	Yes	+	IV	IV	120	II	No
21	Pardatscher et al., 1979 [8]	41	M	No	T2–8	Yes	Yes	Yes	6	Yes	–	IV	III	n.a.	n.a.	n.a.
					T8											n.a.
22	Shalit/Sandbank, 1981 [32]	21	F	No	C2-T2	Yes	Yes	No	6	Yes	+	IV	III	18	II	No
23	Guidetti, 1967 [33], Cantore et al., 1982 [34]	54	F	No	C3–5	Yes	Yes	No	24	Yes	+	II	I	n.a.	n.a.	n.a.
24		57	M	No	T12–L1	No	No	No	n.a.	Yes	+	I	I	n.a.	n.a.	n.a.
25	Lesoin et al., 1983 [35]	45	F	No	C3–7	No	No	No	6	Yes	+	n.a.	II	n.a.	n.a.	n.a.
26		28	M	No	L1	No	Yes	Yes	50	Yes	+	n.a.	III	11	II	No
27	Rout et al., 1983 [36]	50	F	No	C3–5	Yes	Yes	Yes	60	Yes	+	III	III	12	II	No
28	Kang/Song, 1983 [37]	47	M	No	C3–6	Yes	Yes	No	12	Yes	+	IV	III	6	II	No
29	Bouchez et al., 1984 [38]	34	M	No	C2–7	Yes	Yes	No	12	Yes	–	II	II	60	IV	No
30	Drapkin et al., 1985 [39]	30	F	No	C3–5	Yes	Yes	No	46	Yes	+	II	I	20	I	No
31	Lesoin et al., 1986 [40]	75	M	No	T3–6	Yes	Yes	Yes	60	Yes	+	IV	III	6	III	No
32	Maruki et al., 1986 [41]	42	F	No	T7/8	Yes	Yes	No	n.a.	Yes	+	n.a.	n.a.	n.a.	n.a.	n.a.
33	Ross et al., 1986 [4]	67	F	No	C2–T1	Yes	Yes	Yes	48	Yes	+	II	I	6	I	No
34		36	M	No	C4/5	Yes	Yes	No	4	Yes	++	II	I	n.a.	n.a.	n.a.
35	Char/Cross, 1987 [42]	54	M	No	T3/4	Yes	Yes	Yes	1	Yes	–	II	I	0	n.a.	n.a.
36	Garen et al., 1988 [43]	30	F	No	C3–6	Yes	Yes	Yes	24	Yes	+	II	II	n.a.	n.a.	n.a.
37	Hida et al., 1988 [44]	72	F	No	T8/9	Yes	Yes	Yes	132	Yes	n.a.	n.a.	n.a.	n.a.	n.a.	n.a.
38	Okuda et al., 1988 [45]	23	M	No	Med.–C7	Yes	Yes	No	n.a.	Yes	+	IV	III	6	III	No
39	Gorman et al., 1989 [46]	15	F	No	C5/6	Yes	Yes	No	8	Yes	+	II	III	5	II	No
40	Sharma et al., 1989 [47]	10	M	No	C5	Yes	Yes	Yes	12	Yes	+	IV	III	6	II	No
41	Meisel et al., 1990 [48]	36	M	No	T9/10	Yes	Yes	Yes	36	Yes	++	III	II	2	I	No
42	Li/Holtas, 1991 [49]	67	F	n.a.	C2	n.a.	n.a.	n.a.	n.a.	Yes	n.a.	n.a.	n.a.	n.a.	n.a.	n.a.
43	Herregodts et al., 1991 [50]	49	F	No	T2	No	Yes	Yes	60	Yes	+	III	III	2	II	No
44	Jacquet et al., 1992 [51]	44	M	No	T12 – L1	No	No	No	5	Yes	++	I	I	n.a.	n.a.	n.a.
45	Morimoto et al., 1992 [52]	42	M	No	T7–9	No	Yes	No	13	Yes	++	II	I	n.a.	n.a.	n.a.
46	Benini et al., 1993 [53]	40	M	No	T7–9	Yes	Yes	Yes	36	Yes	+	III	IV	5	II	No
47		43	M	No	C5/6	Yes	No	Yes	60	Yes	–	I	IV	12	IV	No
48	Sekerci et al., 1993 [54]	30	F	No	T1–3	Yes	Yes	No	4	Yes	o	II	IV	6	II	No
49	Radhakrishnan et al., 1993 [55]	50	F	No	C2–5	Yes	Yes	No	60	Yes	+	IV	II	12	II	No
50		55	M	No	C4–6	Yes	Yes	No	12	Yes	+	II	II	3	II	No
51	Nicoletti et al., 1994 [56]	47	F	No	C3–5	No	Yes	No	6	Yes	+	V	III	12	n.a.	No
52	Duong et al., 1995 [57]	34	M	No	T5–7	No	Yes	No	18	Yes	++	II	I	60	n.a.	Yes
53		53	F	No	T11–L2	No	Yes	No	24	Yes	–	II	V	36	V	Yes
54	Melancia et al., 1996 [58]	39	F	No	T8	Yes	Yes	No	8	Yes	+	II	n.a.	18	I	No
55	Lee et al., 1996 [2]	31	F	NF 2	C5–T3	n.a.	n.a.	n.a.	n.a.	Yes	n.a.	n.a.	n.a.	12	II	n.a.
56	Bhayani/Goel, 1996 [6]	15	M	NF	C4/5	No	Yes	No	18	Yes	+	III	II	2	I	No
					C5											No
57	Botelho et al., 1996 [59]	52	F	No	C4–6	Yes	Yes	No	48	Yes	+	III	n.a.	12	II	No
58	Innocenzi et al., 1996 [60]	44	M	No	C1–3	No	Yes	No	18	Yes	++	II	II	24	I	No
59	Bekar et al., 1997 [61]	40	M	No	C2–T1	Yes	Yes	Yes	60	Yes	n.a.	II	III	12	III	No
60	Beşkonakli et al., 1997 [62]	42	F	No	T8	Yes	Yes	No	12	Yes	+	III	II	12	II	No
61	Chitoku et al., 1998 [63]	26	M	NF2	T4/5	Yes	Yes	No	n.a.	Yes	o	III	III	n.a.	n.a.	n.a.
62	Kotil et al., 1998 [64]	20	F	NF 2	T10/11	n.a.	n.a.	n.a.	n.a.	Yes	–	n.a.	n.a.	0	n.a.	n.a.
63	Hejazi/Hassler, 1998 [65]	65	M	No	T12–L1	Yes	Yes	Yes	120	Yes	++	n.a.	n.a.	n.a.	n.a.	No
64	Binatli et al., 1999 [66]	9	M	No	C6–T1	Yes	Yes	Yes	4	Yes	++	II	I	3	I	No
65	Arellanes-Chávez et al., 2000 [67]	18	M	No	C2–5	No	Yes	No	36	Yes	+	II	II	n.a.	n.a.	n.a.
66	Riffaud et al., 2000 [3]	25	M	No	C1/2	Yes	Yes	No	12	Yes	+	III	III	12	II	No
67	Ogunbgo et al., 2000 [68]	24	M	No	C4–7	Yes	Yes	No	36	Yes	+	III	n.a.	18	II	No
68	Kodama et al., 2000 [69]	37	F	No	C3–5	Yes	Yes	No	108	Yes	+	n.a.	n.a.	n.a.	n.a.	n.a.
69		17	F	No	C1	Yes	Yes	Yes	12	Yes	+	n.a.	n.a.	n.a.	n.a.	n.a.
70	Patronas et al., 2001 [70]	26	n.a.	NF 2	n.a.	n.a.	n.a.	n.a.	n.a.	Yes	n.a.	n.a.	n.a.	n.a.	n.a.	n.a.
71	Kono et al., 2001 [71]	59	M	No	T2	Yes	Yes	No	6	Yes	+	n.a.	n.a.	n.a.	n.a.	n.a.
72	Maira et al., 2001 [72]	69	M	No	C2	Yes	Yes	Yes	n.a.	Yes	++	III	I	36	n.a.	No
73	Sasaki et al., 2002 [73]	53	M	NF 2	C5/6	Yes	Yes	No	n.a.	Yes	+	II	II	n.a.	n.a.	n.a.
74	Darwish et al., 2002 [74]	68	F	No	C3/4	Yes	Yes	No	108	Yes	o	II	II	n.a.	n.a.	n.a.
75	Brown et al., 2002 [75]	51	F	No	T3–8	Yes	Yes	No	24	Yes	+	III	IV	6	III	No
76	O’Brien et al., 2003 [76]	48	M	No	T11–L1	Yes	Yes	No	6	Yes	++	I	I	6	I	No
77	Colosimo et al., 2003 [77]	59	M	No	C2	Yes	Yes	No	12	Yes	++	n.a.	n.a.	48	I	No
78		47	F	No	T8	No	Yes	No	12	Yes	+	n.a.	III	36	II	No
79	Panagiotopoulos et al., 2004 [78]	71	M	No	T6	Yes	Yes	No	12	Yes	++	IV	II	36	I	No
80		51	M	No	T9/10	Yes	Yes	No	3	Yes	+	IV	II	n.a.	n.a.	n.a.
81	Siddiqui/Shah, 2004 [79]	13	F	NF 2	Med.–C3	Yes	Yes	No	6	Yes	+	III	n.a.	3	II	No
82	Conti et al., 2004 [80]	28	F	NF 2	C1	Yes	Yes	Yes	n.a.	Yes	n.a.	IV	n.a.	n.a.	n.a.	n.a.
83		31	F	No	C4–6	n.a.	n.a.	n.a.	72	Yes	+	n.a.	n.a.	n.a.	n.a.	Yes
84		44	M	No	T10	n.a.	n.a.	n.a.	36	Yes	+	n.a.	n.a.	n.a.	n.a.	No
85	Chavez-Lopez et al., 2004 [81]	40	M	No	C4–6	Yes	Yes	No	24	Yes	+	II	I	n.a.	n.a.	n.a.
86	El Malki et al., 2005 [82]	40	F	No	C1–6	Yes	Yes	No	84	Yes	+	n.a.	n.a.	6	n.a.	No
87	Amato et al., 2005 [83]	38	F	No	C4	Yes	No	No	1	Yes	+	n.a.	n.a.	36	I	No
88	Matsuyama et al., 2009 [84], Kim et al., 2005 [85]	72	F	No	T8/9	Yes	Yes	No	10	Yes	+	II	II	n.a.	n.a.	n.a.
89	Kyoshima et al., 2005 [86]	54	M	No	T9/10	Yes	Yes	Yes	48	Yes	+	II	III	60	II	No
90	Shenoy/Raja, 2005 [87]	29	M	No	C4–7	Yes	Yes	Yes	36	Yes	+	n.a.	n.a.	n.a.	n.a.	n.a.
91	Kahilogullari et al., 2005 [88]	38	F	No	T12–L2	Yes	No	No	7	Yes	++	I	I	n.a.	n.a.	n.a.
92	Ho et al., 2006 [89]	45	M	No	C5/6	No	No	No	n.a.	Yes	+	I	I	4	I	No
93	Mukerji et al., 2007 [90]	8	M	No	C5–7	Yes	Yes	Yes	6	Yes	+	V	n.a.	18	I	No
94	Hida et al., 2008 [91]	41	M	No	C1/2	Yes	Yes	Yes	6	Yes	+	n.a.	n.a.	n.a.	n.a.	n.a.
95		30	M	No	C5–7	Yes	Yes	No	n.a.	Yes	+	n.a.	n.a.	n.a.	n.a.	n.a.
96	Kim et al., 2009 [92]	11	F	No	T5/6	Yes	Yes	Yes	9	Yes	–	II	IV	138	III	No
97	Nicácio et al., 2009 [93]	40	M	No	C4–6	Yes	Yes	Yes	24	Yes	+	III	III	24	III	No
98	Hayashi et al., 2009 [94]	78	F	No	T11–L1	Yes	Yes	No	240	Yes	o	II	III	10	III	No
99	Ohtonari et al., 2009 [95]	29	M	No	T12–L1	No	Yes	Yes	8	Yes	++	II	I	n.a.	n.a.	n.a.
100	Adam et al., 2010 [96]	21	F	No	C2–5	Yes	Yes	No	18	Yes	++	II	I	12	I	No
101		46	F	No	T2–6	n.a.	n.a.	n.a.	6	Yes	+	III	n.a.	48	n.a.	No
102	Lyle et al., 2010 [97]	0	M	No	T2–Sacr.	Yes	Yes	n.a.	n.a.	Yes	n.a.	n.a.	n.a.	n.a.	n.a.	n.a.
103	Bernal-García et al., 2010 [5]	35	F	No	T1–5	Yes	Yes	Yes	36	Yes	+	III	n.a.	204	II	No
104		18	F	NF 2	C5–7	Yes	Yes	No	24	Yes	+	III	n.a.	n.a.	II	No
					Med.–C5											No
105	Teo et al., 2011 [98]	44	M	No	C5/6	Yes	Yes	No	24	Yes	+	II	I	n.a.	n.a.	n.a.
106	Ryu et al., 2011 [99]	68	M	No	T6/7	Yes	Yes	No	17	Yes	+	III	III	1	II	No
107	Vij et al., 2011 [100]	25	M	No	T10/11	Yes	Yes	Yes	36	Yes	–	III	IV	n.a.	n.a.	n.a.
108	Das et al., 2012 [101]	55	M	No	C2/3	No	No	No	n.a.	Yes	n.a.	n.a.	n.a.	n.a.	n.a.	n.a.
109	Li et al., 2013 [102]	42	M	No	T10/11	Yes	Yes	Yes	18	Yes	+	IV	IV	18	I	No
110	Lee et al., 1999 [103], Lee et al., 2013 [104]	39	F	No	C4–7	n.a.	n.a.	n.a.	n.a.	Yes	+	n.a.	n.a.	n.a.	n.a.	No
111		41	F	No	C5/6	n.a.	n.a.	n.a.	n.a.	Yes	+	n.a.	n.a.	n.a.	n.a.	No
112		49	F	No	C5–7	n.a.	n.a.	n.a.	n.a.	Yes	+	n.a.	n.a.	n.a.	n.a.	No
113		46	F	No	T1/2	n.a.	n.a.	n.a.	n.a.	Yes	+	n.a.	n.a.	n.a.	n.a.	No
114		19	F	No	T6–8	n.a.	n.a.	n.a.	n.a.	Yes	+	n.a.	n.a.	n.a.	n.a.	No
115		42	M	No	T7/8	n.a.	n.a.	n.a.	n.a.	Yes	+	n.a.	n.a.	n.a.	n.a.	No
116		60	M	No	T7–10	n.a.	n.a.	n.a.	n.a.	Yes	+	n.a.	n.a.	n.a.	n.a.	No
117		44	M	No	T8/9	n.a.	n.a.	n.a.	n.a.	Yes	+	n.a.	n.a.	n.a.	n.a.	No
118		37	F	No	T9/10	n.a.	n.a.	n.a.	n.a.	Yes	+	n.a.	n.a.	n.a.	n.a.	No
119		78	M	No	T10/11	n.a.	n.a.	n.a.	n.a.	Yes	+	n.a.	n.a.	n.a.	n.a.	No
120	Eljebbouri et al., 2013 [105]	10	M	No	T7–9	Yes	Yes	Yes	6	Yes	+	III	n.a.	18	I	No
121	Wu et al., 2011 [106], Yang et al., 2014 [107]	52	M	No	C6–T4	No	Yes	Yes	120	Yes	o	III	III	154	III	No
122		41	F	No	C4–6	No	Yes	No	6	Yes	++	II	III	140	I	No
123		39	M	No	C3–5	Yes	No	No	12	Yes	++	I	I	125	I	No
124		35	M	No	C6	Yes	No	No	36	Yes	++	I	II	114	I	No
125		46	M	No	T3–5	Yes	Yes	No	12	Yes	+	III	III	102	II	No
126		61	M	No	C6/7	Yes	No	No	24	Yes	++	II	I	94	I	No
127		42	M	No	T10–12	Yes	No	No	24	Yes	++	III	II	85	I	No
138		31	M	No	C3/4	Yes	No	No	12	Yes	++	II	I	78	I	No
129		56	F	No	C5/6	Yes	Yes	No	36	Yes	++	II	III	74	I	No
130		60	F	No	T2/3	Yes	No	No	36	Yes	++	II	I	65	I	No
131		48	M	No	T9/10	Yes	Yes	Yes	144	Yes	+	III	IV	58	III	No
132		59	M	No	C1/2	Yes	No	No	36	Yes	++	I	III	54	I	No
133		50	F	No	C5/T1	Yes	No	No	24	Yes	++	II	III	51	I	No
134		57	M	No	C4–6	No	No	No	6	Yes	++	II	II	47	I	No
135		44	F	No	C5–7	No	Yes	No	48	Yes	++	II	II	41	I	No
136		44	M	No	T3	Yes	Yes	No	12	Yes	++	II	II	24	I	No
137		40	M	No	C3	Yes	No	No	2	Yes	++	II	II	20	I	No
138		34	M	No	T12	No	Yes	No	48	Yes	++	II	II	16	I	No
139		17	M	No	T6–8	Yes	No	No	12	Yes	++	II	III	12	I	No
140		38	M	No	T11	Yes	No	Yes	18	Yes	++	III	II	6	I	No
141	Yang et al., 2015 [108]	35	M	No	T11/12	Yes	Yes	Yes	24	Yes	++	II	II	3	I	No
142	Gupta et al., 2015 [109]	48	M	No	C3/4	Yes	Yes	No	5	Yes	+	III	III	12	II	No
143	Jagannatha et al., 2016 [110]	11	M	No	T11/12	Yes	Yes	Yes	12	Yes	++	III	n.a.	6	n.a.	No
144	Sun et al., 2017 [111]	24	M	n.a.	C1/2	Yes	Yes	Yes	6	Yes	+	II	I	n.a.	n.a.	n.a.
145	Nayak et al., 2017 [112]	28	M	No	T1–9	Yes	Yes	Yes	36	Yes	+	IV	III	n.a.	n.a.	n.a.
146	Gao et al., 2017 [113]	34–59	6 M 2 F	No	T8/9	n.a.	n.a.	n.a.	n.a.	Yes	n.a.	n.a.	n.a.	n.a.	n.a.	n.a.
147				No	T9/10	n.a.	n.a.	n.a.	n.a.	Yes	n.a.	n.a.	n.a.	n.a.	n.a.	n.a.
148				No	T10	n.a.	n.a.	n.a.	n.a.	Yes	n.a.	n.a.	n.a.	n.a.	n.a.	n.a.
149				No	T4–6	n.a.	n.a.	n.a.	n.a.	Yes	n.a.	n.a.	n.a.	n.a.	n.a.	n.a.
150				No	T10/11	n.a.	n.a.	n.a.	n.a.	Yes	n.a.	n.a.	n.a.	n.a.	n.a.	n.a.
151				No	C6–T1	n.a.	n.a.	n.a.	n.a.	Yes	n.a.	n.a.	n.a.	n.a.	n.a.	n.a.
152				No	C5/6	n.a.	n.a.	n.a.	n.a.	Yes	n.a.	n.a.	n.a.	n.a.	n.a.	n.a.
153				No	C4–7	n.a.	n.a.	n.a.	n.a.	Yes	n.a.	n.a.	n.a.	n.a.	n.a.	n.a.
154	Karatay et al., 2017 [114]	30	F	No	T12/L1	No	Yes	No	2	Yes	+	II	n.a.	n.a.	n.a.	n.a.
155	Li et al., 2017 [115]	30	M	No	C3–5	No	No	No	1	No (autopsy)	n.a.	n.a.	n.a.	n.a.	n.a.	n.a.
156	Navarro Fernández et al., 2018 [116]	19	M	No	C6–7	Yes	Yes	No	36	Yes	+	IV	IV	1	III	No
157	Landi et al., 2018 [117]	8	F	No	T10/11	Yes	Yes	No	8	Yes	++	III	II	84	I	No
158	Singh et al., 2018 [118]	27	F	No	T12–L2	Yes	Yes	Yes	12	Yes	+	III	III	6	III	No
159	Wang et al., 2018 [119]	9	M	No	T8	Yes	Yes	No	6	Yes	++	II	I	36	I	No
160	Shi et al., 2019 [120]	42	F	n.a.	Cerv.	n.a.	n.a.	n.a.	n.a.	Yes	n.a.	n.a.	n.a.	36	n.a.	n.a.
161	Dhake/Chatterjee, 2019 [121]	10	M	No	T10–12	Yes	Yes	Yes	6	Yes	+	III	n.a.	216	V	Yes
162		57	F	No	T9/10	Yes	Yes	No	24	Yes	+	IV	n.a.	24	III	Yes
163	Dai et al., 2019 [122]	34	M	No	C3/4	Yes	Yes	No	24	Yes	++	I	I	12	I	No
164	Sekar et al., 2019 [123]	37	F	No	C5–7	Yes	Yes	Yes	12	Yes	n.a.	II	n.a.	n.a.	n.a.	n.a.
165	Kelly et al., 2020 [124]	43	M	No	C4-T2	Yes	Yes	No	18	Yes	+	V	V	3	IV	No

*Modified McCormick scale

*n.a.* information not available

*NF* neurofibromatosis

*OP* operation

We reviewed the included cases with respect to preoperative neurological status, the postoperative outcome and the follow-up, including tumour recurrence. In addition, we calculated the modified McCormick scale to determine the neurological status preoperatively and postoperatively. The analysis of preoperative neurological symptoms showed that sensory disturbance appeared in 67%, motor deficits in 68% and dysfunction of the autonomic nervous system, such as sphincter dysfunction, in 26% of the cases. The main duration of symptoms was 29 months. The preoperative neurological status according to the modified McCormick scale showed the following distribution: grade I (6%), grade II (27%), grade III (21%), grade IV (12%) and grade V (4%); in 30% of the cases, the preoperative modified McCormick scale was not determinable (Table 1).

Our review showed that 161 of 165 patients underwent surgery; in four cases, the diagnosis of IMS was made postmortem by autopsy. The analysis of the postoperative recovery revealed that complete recovery was achieved in 23%, symptom improvement in 51% and stable neurological condition in 4% of the cases. The neurological symptoms worsened in only 4% of cases and in another 4% the patient died after surgery. Information on the postoperative recovery was missing in 14% of the cases. The postoperative neurological status according to the modified McCormick scale showed the following distribution: grade I (16%), grade II (16%), grade III (19%), grade IV (10%) and grade V (3%); in 36% of the cases, the postoperative modified McCormick scale was not determinable (Table 1).

Additionally, we examined the postoperative outcome depending on the duration of symptoms. We defined “long duration of symptoms” as a duration of symptoms for more than 10 years. Patients with IMS and a duration of symptoms of < 10 years recovered completely in 23%, improved in 52% and were in stable neurological condition in 3% of cases; 5% these patients had worsening of symptoms and 4% died after operation. Patients with IMS and a duration of symptoms of ≥ 10 years recovered completely in only 17%, improved in 17% and were in stable neurological condition in 50%; none of these patients had worsening of symptoms or died after operation. Information on the postoperative outcome depending on the duration of symptoms was not determinable in 13% of the patients with a symptom duration < 10 years and in 16% of the patients with a symptom duration of ≥ 10 years.

The average duration of follow-up on a patient with IMS was 34 months. Tumour recurrence was only observed in 4% of the cases (Table 1).

Information on MRI images were available in only half of the cases. In the available T_1_-weighted images, most cases showed an isointense (18.1%) or hypointense (16.9%) imaging pattern; mixed (6.8%) and hyperintense (6.2%) patterns were observed less frequently. T_2_-weighted images showed in 23.2% a hyperintense, in 11.9% an isointense, in 8.5% a mixed and in 7.9% a hypointense pattern. All cases showed a gadolinium enhancement, which was homogenous in 32.8%, heterogenous in 18.6%, some cases showed only a circular (5.6%) and 2 cases were reported to only show minimal gadolinium enhancement (1.1%). 17.5% of the IMS showed a cystic component. Perifocal edema was observed in 22% of the cases; 20.9% of cases were associated with syringomyelia (Table 2).

**Table 2:** Radiological findings

Case No.	Reference	Localization	MRI					
		Vertebra	T1	T2	GA	CYS	OE	SYX
1	Penfield, 1932 [13]	C5	n.a.	n.a.	n.a.	n.a.	n.a.	n.a.
2	Rasmussen et al., 1940 [14]	C4–7	n.a.	n.a.	n.a.	n.a.	n.a.	n.a.
3	Roka, 1951 [15]	Cerv.	n.a.	n.a.	n.a.	n.a.	n.a.	n.a.
4	Rose, 1954 [16]	C5	n.a.	n.a.	n.a.	n.a.	n.a.	n.a.
5	Riggs/Clary, 1957 [17]	C4/5	n.a.	n.a.	n.a.	n.a.	n.a.	n.a.
6	Ramamurthi et al., 1958 [18]	T2	n.a.	n.a.	n.a.	n.a.	n.a.	n.a.
7	Scott/Bentz, 1962 [19]	T3	n.a.	n.a.	n.a.	n.a.	n.a.	n.a.
8	McCormick et al., 1964 [20]	L2	n.a.	n.a.	n.a.	n.a.	n.a.	n.a.
9	Sloof, 1964 [9]	Cerv.	n.a.	n.a.	n.a.	n.a.	n.a.	n.a.
		Cerv.	n.a.	n.a.	n.a.	n.a.	n.a.	n.a.
		Cerv.	n.a.	n.a.	n.a.	n.a.	n.a.	n.a.
10	Mason/Keigher, 1968 [21]	T8–10	n.a.	n.a.	n.a.	n.a.	n.a.	n.a.
11	Chigasaki/Pennybacker, 1968 [22]	T3	n.a.	n.a.	n.a.	n.a.	n.a.	n.a.
12	Van Duinen, 1971 [23]	C3	n.a.	n.a.	n.a.	n.a.	n.a.	n.a.
13	Fabres et al., 1972 [24]	T2/3	n.a.	n.a.	n.a.	n.a.	n.a.	n.a.
14	Cambier et al., 1974 [25]	C2–4	n.a.	n.a.	n.a.	n.a.	n.a.	n.a.
15	Wood et al., 1975 [26]	C3	n.a.	n.a.	n.a.	n.a.	n.a.	n.a.
16	Schmitt, 1975 [27]	L1	n.a.	n.a.	n.a.	n.a.	n.a.	n.a.
17	Isu et al., 1976 [28]	C1	n.a.	n.a.	n.a.	n.a.	n.a.	n.a.
18	Kumar/Gulati, 1977 [29]	Cerv.	n.a.	n.a.	n.a.	n.a.	n.a.	n.a.
		T7–9	n.a.	n.a.	n.a.	n.a.	n.a.	n.a.
19	Vailati et al., 1979 [30]	T8/9	n.a.	n.a.	n.a.	n.a.	n.a.	n.a.
20	Gegalian, 1979 [31]	T10/11	n.a.	n.a.	n.a.	n.a.	n.a.	n.a.
21	Pardatscher et al., 1979 [8]	T2–8	n.a.	n.a.	n.a.	n.a.	n.a.	n.a.
		T8	n.a.	n.a.	n.a.	n.a.	n.a.	n.a.
22	Shalit/Sandbank, 1981 [32]	C2-T2	n.a.	n.a.	n.a.	n.a.	n.a.	n.a.
23	Guidetti, 1967 [33] Cantore et al., 1982 [34]	C3–5	n.a.	n.a.	n.a.	n.a.	n.a.	n.a.
			n.a.	n.a.	n.a.	n.a.	n.a.	n.a.
24		T12 – L1	n.a.	n.a.	n.a.	n.a.	n.a.	n.a.
25	Lesoin et al., 1983 [35]	C3–7	n.a.	n.a.	n.a.	n.a.	n.a.	n.a.
26		L1	n.a.	n.a.	n.a.	n.a.	n.a.	n.a.
27	Rout et al., 1983 [36]	C3–5	n.a.	n.a.	n.a.	n.a.	n.a.	n.a.
28	Kang/Song, 1983 [37]	C3–6	n.a.	n.a.	n.a.	n.a.	n.a.	n.a.
29	Bouchez et al., 1984 [38]	C2–7	n.a.	n.a.	n.a.	n.a.	n.a.	n.a.
30	Drapkin et al., 1985 [39]	C3–5	n.a.	n.a.	n.a.	n.a.	n.a.	n.a.
31	Lesoin et al., 1986 [40]	T3–6	n.a.	n.a.	n.a.	n.a.	n.a.	n.a.
32	Maruki et al., 1986 [41]	T7/8	n.a.	n.a.	n.a.	n.a.	n.a.	n.a.
33	Ross et al., 1986 [4]	C2–T1	Iso.	Hyper.	n.a.	n.a.	n.a.	n.a.
34		C4/5	n.a.	n.a.	n.a.	n.a.	n.a.	n.a.
35	Char/Cross, 1987 [42]	T3/4	n.a.	n.a.	n.a.	n.a.	n.a.	n.a.
36	Garen et al., 1988 [43]	C3–6	n.a.	Hyper.	n.a.	n.a.	n.a.	n.a.
37	Hida et al., 1988 [44]	T8/9	n.a.	n.a.	n.a.	n.a.	n.a.	n.a.
38	Okuda et al., 1988 [45]	Med.–C7	n.a.	n.a.	n.a.	n.a.	n.a.	n.a.
39	Gorman et al., 1989 [46]	C5/6	Mixed	Hyper.	n.a.	No	No	No
40	Sharma et al., 1989 [47]	C5	n.a.	n.a.	n.a.	n.a.	n.a.	n.a.
41	Meisel et al., 1990 [48]	T9/10	Hyper.	Hypo.	Homo.	No	Yes	Yes
42	Li/Holtas, 1991 [49]	C2	Hypo./Iso.	Iso./Hypo.	Homo.	No	Yes	No
43	Herregodts et al., 1991 [50]	T2	Hyper.	n.a.	Homo.	No	Yes	No
44	Jacquet et al., 1992 [51]	T12–L1	n.a.	n.a.	Homo.	n.a.	n.a.	n.a.
45	Morimoto et al., 1992 [52]	T7–9	n.a.	n.a.	n.a.	n.a.	n.a.	n.a.
46	Benini et al., 1993 [53]	T7–9	n.a.	Hyper.	Minimal	No	No	No
47		C5/6	n.a.	Iso.	Homo.	No	No	Yes
48	Sekerci et al., 1993 [54]	T1–3	n.a.	n.a.	n.a.	n.a.	n.a.	n.a.
49	Radhakrishnan et al., 1993 [55]	C2–5	n.a.	n.a.	n.a.	n.a.	n.a.	n.a.
50		C4–6	n.a.	n.a.	n.a.	n.a.	n.a.	n.a.
51	Nicoletti et al., 1994 [56]	C3–5	Hyper.	Hypo.	n.a.	No	No	No
52	Duong et al., 1995 [57]	T5–7	Iso.	Iso.	Homo.	Yes	Yes	Yes
53		T11–L2	n.a.	n.a.	n.a.	n.a.	n.a.	n.a.
54	Melancia et al., 1996 [58]	T8	Hypo.	Hypo.	Homo.	No	No	Yes
55	Lee et al., 1996 [2]	C5–T3	n.a.	n.a.	n.a.	n.a.	n.a.	n.a.
56	Bhayani/Goel, 1996 [6]	C4/5	n.a.	n.a.	Homo.	n.a.	n.a.	n.a.
		C5	n.a.	n.a.	Homo.	n.a.	n.a.	n.a.
57	Botelho et al., 1996 [59]	C4–6	n.a.	n.a.	Homo.	Yes	No	Yes
58	Innocenzi et al., 1996 [60]	C1–3	Hypo.	Hyper.	Homo.	No	No	No
59	Bekar et al., 1997 [61]	C2-T1	Hyper.	Hyper.	Homo.	Yes	No	No
60	Beşkonakli et al., 1997 [62]	T8	Hyper.	n.a.	n.a.	No	Yes	No
61	Chitoku et al., 1998 [63]	T4/5	Hypo.	Iso.	n.a.	n.a.	n.a.	Yes
62	Kotil et al., 1998 [64]	T10/11	n.a.	Hyper.	n.a.	n.a.	n.a.	n.a.
63	Hejazi/Hassler, 1998 [65]	T12–L1	n.a.	n.a.	n.a.	n.a.	n.a.	n.a.
64	Binatli et al., 1999 [66]	C6–T1	n.a.	n.a.	Homo.	n.a.	n.a.	Yes
65	Arellanes-Chávez et al., 2000 [67]	C2–5	Iso.	Hyper.	Homo.	Yes	No	Yes
66	Riffaud et al., 2000 [3]	C1/2	Hyper.	Hypo.	Homo.	No	Yes	No
67	Ogunbgo et al., 2000 [68]	C4–7	n.a.	n.a.	Heter.	No	No	Yes
68	Kodama et al., 2000 [69]	C3–5	Hyper.	Iso./Hypo.	Homo.	No	Yes	No
69		C1	Hypo.	Hyper.	Circ.	Yes	Yes	Yes
70	Patronas et al., 2001 [70]	n.a.	n.a.	n.a.	n.a.	n.a.	n.a.	n.a.
71	Kono et al., 2001 [71]	T2	Iso.	Iso./Hyper.	Homo.	Yes	Yes	No
72	Maira et al., 2001 [72]	C2	n.a.	n.a.	Homo.	No	No	No
73	Sasaki et al., 2002 [73]	C5/6	Hypo.	Iso.	n.a.	n.a.	n.a.	n.a.
74	Darwish et al., 2002 [74]	C3/4	n.a.	n.a.	Homo.	No	No	No
75	Brown et al., 2002 [75]	T3–8	n.a.	n.a.	Heter.	No	No	Yes
76	O’Brien et al., 2003 [76]	T11–L1	n.a.	Hyper.	n.a.	Yes	No	No
77	Colosimo et al., 2003 [77]	C2	Iso.	Hypo.	Homo.	No	Yes	No
78		T8	Iso.	Hyper.	Homo.	Yes	Yes	No
79	Panagiotopoulos et al., 2004 [78]	T6	Hypo.	Hyper.	Homo.	No	No	No
80		T9/10	Hypo.	Hyper.	Homo.	Yes	No	No
81	Siddiqui/Shah, 2004 [79]	Med.–C3	Iso/Hypo.	Hyper.	Heter.	No	No	Yes
82	Conti et al., 2004 [80]	C1	n.a.	n.a.	n.a.	n.a.	n.a.	n.a.
83		C4–6	n.a.	n.a.	n.a.	n.a.	n.a.	n.a.
84		T10	n.a.	n.a.	n.a.	n.a.	n.a.	n.a.
85	Chavez-Lopez et al., 2004 [81]	C4–6	Iso.	Iso./Hyper.	Homo.	No	Yes	No
86	El Malki et al., 2005 [82]	C1–6	Hyper.	Hyper.	Heter.	Yes	No	Yes
87	Amato et al., 2005 [83]	C4	Hyper.	n.a.	Homo.	No	No	Yes
88	Matsuyama et al., 2009 [84] Kim et al., 2005 [85]	T8/9	n.a.	Iso.	Homo.	No	Yes	No
89	Kyoshima et al., 2005 [86]	T9/10	Iso./Hypo.	Iso.	Circ.	No	No	No
90	Shenoy/Raja, 2005 [87]	C4–7	Iso./Hypo.	Hyper.	Circ.	No	No	Yes
91	Kahilogullari et al., 2005 [88]	T12–L2	n.a.	n.a.	Heter.	n.a.	n.a.	n.a.
92	Ho et al., 2006 [89]	C5/6	Iso.	Hyper.	Homo.	No	No	No
93	Mukerji et al., 2007 [90]	C5–7	Iso.	Hyper.	n.a.	No	Yes	No
94	Hida et al., 2008 [91]	C1/2	Hypo.	Iso.	Heter.	No	Yes	No
95		C5–7	n.a.	n.a.	Homo.	No	No	No
96	Kim et al., 2009 [92]	T5/6	Hypo.	Iso.	Circ.	No	No	Yes
97	Nicácio et al., 2009 [93]	C4–6	Hyper.	Hypo.	Heter.	No	Yes	Yes
98	Hayashi et al., 2009 [94]	T11–L1	Hypo.	Iso.	Circ.	Yes	Yes	No
99	Ohtonari et al., 2009 [95]	T12–L1	Iso.	n.a.	Homo.	Yes	No	No
100	Adam et al., 2010 [96]	C2–5	n.a.	n.a.	n.a.	n.a.	n.a.	n.a.
101		T2–6	n.a.	n.a.	n.a.	n.a.	n.a.	n.a.
102	Lyle et al., 2010 [97]	T2–Sacr.	n.a.	Iso.	Heter.	No	No	No
103	Bernal-García et al., 2010 [5]	T1–5	Iso.	Hyper.	Homo.	No	Yes	No
104		C5–7	Hyper.	Iso.	Homo	No	No	No
		Med.–C5	n.a.	n.a.	n.a.	Yes	No	No
105	Teo et al., 2011 [98]	C5/6	Hypo.	Hyper.	Homo.	Yes	Yes	No
106	Ryu et al., 2011 [99]	T6/7	Iso.	Hyper.	Homo.	No	Yes	Yes
107	Vij et al., 2011 [100]	T10/11	Hypo.	Iso.	n.a.	No	No	No
108	Das et al., 2012 [101]	C2/3	Hypo.	Hyper.	n.a.	No	No	Yes
109	Li et al., 2013 [102]	T10/11	Iso.	Hypo.	Heter.	No	Yes	No
110	Lee et al., 1999 [103], Lee et al., 2013 [104]	C4–7	n.a.	n.a.	Heter.	n.a.	n.a.	n.a.
111		C5/6	n.a.	n.a.	Homo.	n.a.	n.a.	n.a.
112		C5–7	n.a.	n.a.	Homo.	n.a.	n.a.	n.a.
113		T1/2	n.a.	n.a.	Homo.	n.a.	n.a.	n.a.
114		T6–8	n.a.	n.a.	Homo.	n.a.	n.a.	n.a.
115		T7/8	n.a.	n.a.	Circ.	n.a.	n.a.	n.a.
116		T7–10	n.a.	n.a.	Heter.	n.a.	n.a.	n.a.
117		T8/9	n.a.	n.a.	Circ.	n.a.	n.a.	n.a.
118		T9/10	n.a.	n.a.	Homo.	n.a.	n.a.	n.a.
119		T10/11	n.a.	n.a.	Homo.	n.a.	n.a.	n.a.
120	Eljebbouri et al., 2013 [105]	T7–9	n.a.	Hyper.	Heter.	Yes	Yes	No
121	Wu et al., 2011 [106], Yang et al., 2014 [107]	C6-T4	Hypo./Iso.	Hyper.	Heter.	Yes	Yes	No
122		C4–6	Hypo.	Hyper.	Homo.	No	No	Yes
123		C3–5	Iso.	Iso.	Homo.	No	No	Yes
124		C6	Hypo.	Hyper.	Homo.	No	No	Yes
125		T3–5	Hypo./Iso.	Hyper.	Heter.	Yes	Yes	No
126		C6/7	Hypo.	Hyper./Iso.	Circ.	Yes	No	No
127		T10–12	Hypo./Iso.	Hyper./Iso.	Heter.	Yes	No	No
138		C3/4	Iso.	Iso.	Heter.	No	No	No
129		C5/6	Hypo.	Hyper.	Heter.	Yes	Yes	No
130		T2/3	Iso.	Iso.	Homo.	No	No	Yes
131		T9/10	Iso.	Hyper.	Homo.	No	No	Yes
132		C1/2	Iso.	Iso.	Homo.	No	No	Yes
133		C5/T1	Hypo.	Hyper./Iso.	Heter.	Yes	Yes	No
134		C4–6	Hypo./Iso.	Hyper.	Heter.	Yes	Yes	No
135		C5–7	Iso.	Hyper./Iso.	Heter.	No	No	Yes
136		T3	Iso.	Iso.	Homo.	No	Yes	No
137		C3	Iso.	Hyper.	Heter.	No	No	Yes
138		T12	Iso.	Hyper./Iso.	Heter.	Yes	No	Yes
139		T6–8	Iso.	Hyper./Iso.	Heter.	Yes	No	Yes
140		T11	Iso.	Iso.	Homo.	No	No	No
141	Yang et al., 2015 [108]	T11/12	Iso.	Hypo.	Heter.	Yes	No	Yes
142	Gupta et al., 2015 [109]	C3/4	n.a.	Iso.	Heter.	Yes	Yes	No
143	Jagannatha et al., 2016 [110]	T11/12	Hyper./Hypo.	Hypo.	Heter.	Yes	No	Yes
144	Sun et al., 2017 [111]	C1/2	Iso.	Iso.	Homo.	No	No	Yes
145	Nayak et al., 2017 [112]	T1–9	Hypo.	Hyper.	Homo.	Yes	No	No
146	Gao et al., 2017 [113]	T8/9	Iso.	Hypo./Hyper.	Heter.	No	Yes	No
147		T9/10	Hypo.	Hypo.	Heter.	No	Yes	No
148		T10	Iso.	Hypo.	Heter.	No	Yes	Yes
149		T4–6	Hypo.	Hyper.	Homo.	No	No	No
150		T10/11	Hypo.	Hypo.	Homo.	No	Yes	No
151		C6–T1	Hypo.	Hypo./Hyper.	Homo.	No	Yes	Yes
152		C5/6	Hypo.	Hypo./Hyper.	Homo.	No	Yes	No
153		C4–7	Hypo.	Hypo./Hyper.	Homo.	No	No	No
154	Karatay et al., 2017 [114]	T12/L1	Hypo.	Hyper.	Homo.	No	No	Yes
155	Li et al., 2017 [115]	C3–5	n.a.	n.a.	n.a.	n.a.	n.a.	n.a.
156	Navarro Fernández et al., 2018 [116]	C6–7	Iso.	Hyper.	Circ.	Yes	Yes	No
157	Landi et al., 2018 [117]	T10/11	n.a.	Hypo.	Homo.	No	No	No
158	Singh et al., 2018 [118]	T12–L2	Hypo./Hyper.	Hyper.	Heter.	Yes	No	No
159	Wang et al., 2018 [119]	T8	Hypo.	iso	Homo.	No	No	Yes
160	Shi et al., 2019 [120]	Cerv.	n.a.	n.a.	n.a.	n.a.	n.a.	n.a.
161	Dhake/Chatterjee, 2019 [121]	T10–12	Iso./Hypo.	Hyper.	Heter.	No	No	No
162		T9/10	Hypo.	Hyper.	Circ.	No	No	No
163	Dai et al., 2019 [122]	C3/4	Iso.	Hyper.	Minimal	No	Yes	No
164	Sekar et al., 2019 [123]	C5–7	Hypo.	Hyper.	n.a.	Yes	Yes	No
165	Kelly et al., 2020 [124]	C4–T2	Iso./Hypo.	Hyper.	Heter.	No	No	Yes

*MRI* magnetic resonance imaging

*T1* T_1_-weighted images

*T2* T_2_-weighted images

*GA* gadolinium enhanced

*CYS* cystic lesion

*OE* oedema in T_2_-weighted images

*SYX* tumour-associated syringomyelia

*iso.* isointense

*Hypo.* hypointense

*Hyper.* hyperintense

*Homo.* homogenous

*Heter.* heterogenous

*Circ.* ciruclar

*n.a.* information not available

## Discussion

To our knowledge, no complete review of all reported cases has been performed thus far. Here, we attempted to gather all reported cases since 1932. Interestingly, we found more cases than previously described in other series [62, 80, 98]. Due to the language barrier, reports in Japanese, Chinese, French, Portuguese, German and Spanish were not included in previous reports. Additionally, keyword research in the known databases did not show all cases; further analysis of reported case series revealed cases, which were missed by keyword research of the databases. This series of 166 cases including our own study is the largest review of cases on IMS. An uncomplete review of this very rare pathology might constitute a limitation, which impacts the estimated epidemiology.

IMS represent 0.3–1.5% of all spinal schwannomas [2–4]. Several studies described a gender distribution of 3:1 (male:female) [93, 107, 113]. Our results showed a higher rate of female patients and thus a gender distribution of 3:2 (male:female). Previous studies found the mean age of disease presentation to be in the fourth decade of life [92, 113, 117]. The mean age of disease presentation in our series was 40.2 years (range: 1 day–78 years old). Thus, the analysis of our series confirmed the previously reported results. The cervical spine followed by the thoracic spine was reported as the most common localization of IMS [3, 85, 88, 89]. These findings are also consistent with our analysis.

Previous studies addressing the clinical features and surgical outcome of patients with IMS revealed sensory disturbance as the most common initial symptom [107]. Our results show that patients with IMS suffer from sensory deficits as often as from motor deficits, but we agree with Yang et al. on the value of sphincter dysfunction as a late symptom [107]. Overall, patients with IMS seem to benefit from operation, which is clearly shown by an improved postoperative neurological status in 86% of the patients. Previous studies on IMS observed that patients with a longer symptom duration benefit less from surgery due to chronical compression of the neuronal tissue by the tumour [107]. In our review, we were not able to confirm this hypothesis, since the analysis of the postoperative outcome as a function of the duration of symptoms revealed no significantly worse outcome for patients with a symptom duration ≥ 10 years. In most of the cases, gross total resection can be achieved easily [107]. In cases in which the tumour is strongly adherent to the surrounding neuronal tissue, subtotal resection should be considered in order to avoid deterioration of the neurological status. In particularly complicated cases, two-stage surgery provides a possible approach towards better therapeutical results [91].

Conti et al. stated that IMS associates with NF; however, several studies showed a prevalence of 0–2% in spinal tumours [7, 70, 80, 103, 125]. Our review found NF in 11 of 166 cases (6.6%). These results reveal slightly higher rates of NF in patients with IMS than previously described; however, no firm association between NF and IMS was found.

IMS are frequently misdiagnosed as another tumour entity because of the tumour location and its heterogenous appearance in MRI diagnostics [113, 122]. Several series described the MRI appearance of schwannomas as being iso/hypointense in the T_1_- and hyperintense in the T_2_-weighted images [1]. However, the T_1_- and T_2_-weighted appearance of IMS varies among studies [107, 113]. The summary of these studies in our review reveals that in most cases, IMS show a similar MRI appearance as schwannomas. Specifically, in T_1_-weighted images, 35% of all cases appeared iso- or hypointense and in T_2_-weighted images, 23.2% were hyperintense. Interestingly, 1/5 of all cases associated with syringomyelia and in 20%, a perilesional edema was observed. The treated patient in our institution suffered from a perilesional edema, which showed a complete remission in the follow-up MRI after 4 months.

The pathogenesis of IMS is controversially debated among experts because of the absence of Schwann cells within the central nervous system (CNS) in healthy individuals [69]. Currently, there are six hypotheses regarding the origin of IMS: (a) conversion of pial mesodermal cells into neuroectodermal Schwann cells [126]; (b) migration and late neoplastic growth of ectopic Schwann cells during embryonal development [18, 30]; (c) origin from Schwann cells from the perivascular nerve plexus surrounding the blood vessels within the CNS [17, 27, 36, 127, 128]; (d) schwannosis in proximity to the anterior spinal artery [129]; (e) centripetal growth from a dorsal nerve root entry zone into the spinal cord [20, 21, 26, 128] and (f) result from imperfect regeneration of the spinal cord after mechanical trauma or chronic disease [130].

Although some association of proliferating vessels around the tumour [4, 32, 35, 68, 102], tumour connection to a nerve root [4, 27, 34, 43, 46, 52, 58, 68, 71, 76, 77, 84, 89, 99, 104, 107, 109, 115, 123] or chronic disease of the spinal cord could be observed in reported cases [39, 100, 107], it is still not possible to make a general statement regarding the pathogenesis of IMS. In our case, a tumour connection to the nerve root could be observed in the MRI of the cervical spine. This is why we rather support the hypothesis of centripetal growth from a nerve root entry zone into the spinal cord as a possible pathomechanism for development of IMS. However, this mechanism is not able to explain the formation of multiple IMS. The special subgroup of multiple IMS might have implications for the pathomechanism of IMS, but the available information do not allow a conclusions about differences in the pathogenesis of singular and multiple IMS.

As part of the preoperative examination and consultation of patients with intramedullary tumours, it is important to make a correct tentative diagnosis to ensure the best possible treatment. Since IMS are benign tumours of the spinal cord, their treatment might differ from other tumours, like spinal astrocytoma or ependymoma. Patients with IMS show a low rate of tumour recurrence. Even in cases with subtotal tumour resection, tumour recurrence is not necessarily observed [107]. In contrast, for patients with spinal ependymoma, the gross total resection is the gold standard to achieve the longest possible progression-free survival [131–134]. Therefore, complete removal of the tumour should be the goal of the surgery. Furthermore, it is unclear if patients with spinal astrocytoma benefit from gross total resection as patients with spinal ependymoma do [135–138]. Additionally, gross total resection is difficult to achieve in patients with spinal astrocytoma without causing a worse neurological outcome, which is why the primary goal of surgery is to spare the surrounding nervous tissue [139, 140]. Unfortunately, spinal astrocytoma and ependymoma are difficult to distinguish from IMS by use of MRI [107, 113, 141]. Therefore, it seems to be important to differentiate intramedullary tumours during surgery with the aid of intraoperative frozen sections in order to provide the patient with the best possible therapy [95, 104].

## Conclusion

IMS are rare tumours of the spinal cord. One hundred sixty-six cases have been reported so far, including the here reported case. IMS are more frequently found in male patients; the mean age of disease presentation is the fourth decade of life. The most common localization of IMS is the cervical spine, followed by the thoracic spine. Although several explanations regarding the pathogenesis of IMS have been proposed, it is still not possible to make a general statement regarding the pathogenesis of these tumours, especially for the subgroup of patients with multiple IMS. In our study, no firm association between NF and IMS was found.

Patients suffering from IMS present in most of the cases with sensory and motor deficits; sphincter dysfunction seems to be a late symptom. Due to heterogenous imaging patterns in MRI, it is difficult to preoperatively differentiate an IMS from other intramedullary tumours. Therefore, intraoperative frozen section might be useful to determine the tumour entity and the best suited surgical strategy. Overall, patients with IMS seem to benefit from operation; in most of the cases, gross total resection can be achieved easily. Nevertheless, further multicentre studies are necessary to elucidate the pathomechanism leading to IMS formation and to determine strategies for the best clinical care for these patients.

## Data Availability

The authors declare that the data supporting the findings of this study are available within the article.
